# Adaptive working memory strategy training in early Alzheimer's disease: randomised controlled trial

**DOI:** 10.1192/bjp.bp.116.182048

**Published:** 2017-01

**Authors:** J. D. Huntley, A. Hampshire, D. Bor, A. Owen, R. J. Howard

**Affiliations:** **J. D. Huntley**, PhD, Institute of Psychiatry, Psychology and Neuroscience, King's College London, London, UK; **A. Hampshire**, PhD, Division of Brain Sciences, Imperial College London, London, UK; **D. Bor**, PhD, Sackler Centre for Consciousness Science, University of Sussex, Brighton; **A. Owen**, PhD, Brain and Mind Institute, University of Western Ontario, London, Ontario, Canada; **R. J. Howard**, MD, Division of Psychiatry, University College London, London, UK

## Abstract

**Background**

Interventions that improve cognitive function in Alzheimer's disease are urgently required.

**Aims**

To assess whether a novel cognitive training paradigm based on ‘chunking’ improves working memory and general cognitive function, and is associated with reorganisation of functional activity in prefrontal and parietal cortices (trial registration: ISRCTN43007027).

**Method**

Thirty patients with mild Alzheimer's disease were randomly allocated to receive 18 sessions of 30 min of either adaptive chunking training or an active control intervention over approximately 8 weeks. Pre- and post-intervention functional magnetic resonance imaging (fMRI) scans were also conducted.

**Results**

Adaptive chunking training led to significant improvements in verbal working memory and untrained clinical measures of general cognitive function. Further, fMRI revealed a bilateral reduction in task-related lateral prefrontal and parietal cortex activation in the training group compared with controls.

**Conclusions**

Chunking-based cognitive training is a simple and potentially scalable intervention to improve cognitive function in early Alzheimer's disease.

Alzheimer's disease, the most common form of dementia, is characterised by progressive impairment in multiple cognitive domains, including episodic memory and working memory.^1^ Working memory capacity is limited to only a few items of information,^2^ consequently humans use executive strategies, such as chunking, to enable working memory to hold complex mental representations. Chunking refers to the process of recognising or enforcing patterns upon information, and compressing it into a more efficient state, thereby creating complex ‘chunks’ of information that can be held within the limited capacity working space of working memory. The ability to use chunking is preserved in the early stages of Alzheimer's disease,^1^ potentially providing a promising target for effective cognitive training in Alzheimer's disease.^3^ Cognitive training involves the use of theoretically driven exercises targeting specific cognitive domains in order to optimise cognitive function.^4^ Cognitive training can lead to improvements in the cognitive tasks and domains specifically trained in healthy people,^5^ and there is growing evidence that working memory training can lead to generalised improvements in non-trained tasks,^6^ particularly tasks that depend on working memory and the control of attention.^7^ Evidence for the efficacy of working memory training in Alzheimer's disease, however, has so far been limited.^8^ Furthermore, cognitive training studies in Alzheimer's disease rarely apply the rigorous control interventions required for a formal clinical trial.^9^

Successful generalisation of cognitive training benefits to non-trained tasks may have a basis in altered processing within ‘domain general’ neural systems that make a broad contribution to cognition. Animal and human studies have demonstrated that encoding, storage and retrieval of information in working memory is associated with activity in the prefrontal cortex (PFC) and posterior parietal cortex (PPC).^10,11^ Several groups have identified activation in the PFC and left PPC accompanying the executive control of information within verbal working memory.^12^ This network is also associated with a range of higher-level executive processes,^13^ including the successful use of chunking strategies.^14,15^ Consequently, effective cognitive training may be associated with training-induced plasticity in this common prefrontal–parietal network^7^ and chunking has been postulated as a major strategy underlying these successful cognitive training regimes.^16^ We conducted a parallel randomised controlled trial to investigate whether training individuals with early Alzheimer's disease using an adaptive chunking working memory task would improve their working memory capacity. We hypothesised that training-related improvements in working memory capacity would generalise across different modalities of working memory tasks, as well as measures of general cognitive functioning and executive function, and that these improvements would be accompanied by evidence of plasticity of functional activity in the PFC and parietal cortex.

## Method

A total of 30 participants with early Alzheimer's disease (according to NINCDS-ADRDA criteria^17^) were recruited from memory services of the South London and Maudsley NHS Foundation Trust. Of these, 27 had diagnoses of probable Alzheimer's disease and 3 had diagnoses of possible Alzheimer's disease, having recently converted from mild cognitive impairment to Alzheimer's disease. Diagnoses were made by experienced old age psychiatrists unconnected to the study. Inclusion criteria were a Mini-Mental State Examination (MMSE) score of >22/30 and age >60 years (see online supplement DS1 for further details). Exclusion criteria included coexistent neurological or psychiatric disease, substance misuse or significant auditory or visual impairment. All participants had capacity to provide written informed consent to participate in the study, which was approved by the NRES Committee East of England-Cambridge East (REC reference number 10/H0304/68) and registered prior to the onset of the study (trial number: ISRCTN43007027). Following informed consent and baseline functional magnetic resonance imaging (fMRI), participants were randomised to either training (*n* = 15) or control groups (*n* = 15) using an online block randomisation program.

### Outcome measures

The primary outcome measure was a computerised verbal working memory span task, involving both structured (chunkable) and random sequences (see online Fig. DS1). Structured trials consisted of digits presented in runs of consecutive numbers or numbers increasing or decreasing in 2s or 3s (for example, 2,4,6,8 or 9,7,5,1,2,3). Previous studies have demonstrated that structured trials significantly encourage chunking, lessening working memory demand and significantly improving working memory performance.^14,15^ Near transfer of training effects to untrained working memory tasks was assessed using a spatial span task. Both structured and random versions of the task were used, with structured trials consisting of consecutive blocks presented in the same row or column, or in recognisable shapes. In the random version of these tasks, number sequences or blocks were presented in random combinations.

Transfer of training effects to clinical measures of general cognitive function were examined using the MMSE and Alzheimer's Disease Assessment Scale – Cognitive Section (ADAS-Cog). Transfer of training effects to episodic memory was assessed using the Logical Memory II task and Paired Associates Learning task (PAL). Transfer of training effects to executive function was assessed using a verbal fluency task, trail making tasks A and B and computerised grammatical reasoning, ‘odd one out’ and ‘self ordered search’, tasks. See online supplement DS1 for descriptions of all tasks and statistical analyses.

### fMRI

All participants underwent pre- and post-intervention fMRI on a Siemens 3T scanner. While undergoing fMRI, participants performed a five-digit span working memory task adapted for people with Alzheimer's disease from a previous fMRI study in young healthy individuals,^15^ requiring them to encode, retain and then verbally recall the five digits in order. Three blocks of twenty trials were performed and structured or random span sequences were presented pseudo-randomly. See online supplement DS1 for details of fMRI acquisition and analysis.

### Interventions

Participants randomised to the training group underwent 18 sessions of training over approximately 8 weeks, in line with recent studies demonstrating effective cognitive training interventions.^16^ Each session consisted of 30 trials of an adaptive structured digit span task. The initial span length was a three-digit sequence, presented on a computer screen. If the participant correctly recalled the sequence, then the number of digits to be recalled (span) would increase by one for the subsequent trial. Conversely, if the sequence was incorrectly recalled, the next trial would have one fewer digits. In this way participants reached and then oscillated around their maximum span, which could adapt to performance both within and across training sessions. Control participants underwent 18 sessions of an active control intervention involving 30 trials of a fixed, non-adaptive unstructured three-digit span task. This controlled for most aspects of the experimental intervention, apart from the adaptive chunking elements.

## Results

The study was conducted between February 2011 and August 2014. All participants completed the study (see online Fig. DS2). Analysis of baseline demographic information demonstrated no significant differences between the groups on any of the demographic or screening variables (see online Table DS1). The mean or median scores and standard deviations or interquartile ranges for each group at pre- and post-intervention, and effect sizes for all primary and secondary behavioural outcome measures are shown in Table 1.

**Table 1:** Pre- and post-scores and effect sizes of all cognitive outcomes^a^

	Training group (*n* = 15)		Control group (*n* =15)		Effect size, *r*	
	Pre	Post	Pre	Post		*P*
Working memory, mean (s.d.)						
Digit span structured trial	5.49 (0.92)	6.30 (0.90)	5.53 (0.90)	5.79 (0.75)	0.42	**0.017**
Digit span random trial	5.23 (0.84)	5.64 (0.85)	5.01 (0.88)	5.33 (0.69)	0.08	0.670
Spatial span structured trial	3.83 (1.05)	3.98 (0.66)	3.90 (0.70)	4.04 (0.82)	0.01	0.959
Spatial span random trial	3.62 (0.91)	3.74 (0.59)	3.56 (0.75)	3.74 (0.85)	−0.05	0.777

General cognitive function						
Mini-Mental State Examination, mean (s.d.)	26.00 (2.30)	26.10 (2.00)	25.93 (2.09)	24.60 (1.84)	0.44	**0.011**
ADAS-Cog, median (IQR)^b^	11.00 (9.66–18.33)	8.67 (6.33–15.33)	13.00 (9.66–17.66)	14.66 (13–15)	−0.58	**0.001**

Episodic memory, mean (s.d.)						
Logical Memory Task 2	7.20 (8.20)	12.47 (8.27)	7.93 (7.05)	7.73 (8.06)	0.51	**0.003**
Paired Associates Learning task, median (IQR)	3.00 (3–4)	3.00 (3–3)	3.00 (3–3)	3.00 (2–4)	−0.33	0.075

Executive function						
Verbal fluency, mean (s.d.)	8.64 (2.73)	8.00 (2.59)	8.21 (2.52)^c^	8.27 (2.43)	−0.11	0.577
Grammatical reasoning, mean (s.d.)	6.00 (5.28)	5.40 (4.40)	4.73 (4.59)	6.80 (5.72)	−0.32	0.074
Odd one out, mean (s.d.)	10.20 (3.10)	9.40 (3.42)	7.60 (2.29)	8.53 (3.02)	−0.25	0.162
Self ordered search, median (IQR)	4.00 (4, 6)	5.00 (4, 6)	4.00 (3, 5)	5.00 (4, 6)	−0.28	0.121
Trail making task part A, median (IQR)	52 (33–115)	66 (38.4 1–99.00)	63.50 (44.75–112.25)^c^	65.5 (41.25–96.25)	−0.11	0.556

a. Scores for Alzheimer's Disease Assessment Scale – Cognitive section (ADAS-Cog), Paired Associates Learning task, self ordered search task and trail making task part A are shown as medians and interquartile ranges (IQR), as data were not normally distributed. The units are maximum scores for all tasks except trails A (time in seconds), fluency (maximum of 14), Logical Memory Task 2 (maximum of 32). Results of trail making task, part B are not reported due to floor effects at both time points. Results in bold are significant.

b. The ADAS-Cog is inversely scored, therefore higher scores represent more impairment.

c. *n* = 14.

**Fig. 1:**
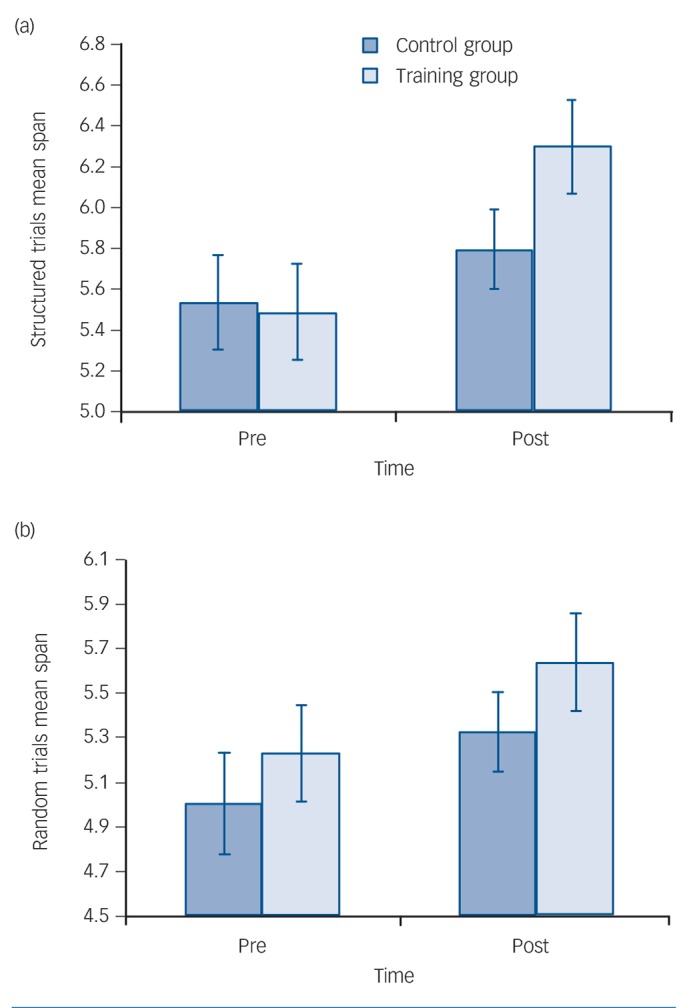
Mean digit span scores on structured and random trials. (a) Mean digit span score on structured trials at pre- and post-intervention; (b) mean digit span scores on random trials at pre- and post-intervention. When each trial type is examined separately there is a significant main effect of time for both trial types (*P*<0.001) and a significant time × group interaction (F(1,28) = 6.40, *P* = 0.017) for structured trials (a), but not for random trials (*P* = 0.67) (b). Error bars are standard errors of mean.

The primary outcomes were the mean digit span scores on structured and random trials (Fig. 1). Repeated measures ANOVA with PrePost (pre *v.* post) and chunking (structured *v.* random trials) as within-participants factors and group as the between-participants factor, revealed a significant main effect of PrePost (*F*(1,28) = 26.282, *P*<0.001), indicating that both groups improved on the digit span task over the course of the study, and a main effect of chunking (*F*(1,28) = 58.605, *P*<0.001), demonstrating that both groups performed significantly better on structured compared with random trials (Fig. 1). The interaction between PrePost, chunking and group neared significance (*F*(1,28) = 4.067, *P* = 0.053). The basis of this complex interaction was examined by performing separate repeated measures ANOVAs for each trial type, with PrePost as the within-participants factor and group as the between-participants factor.

Analysis of the structured trials revealed a significant main effect of PrePost (*F*(1,28) = 24.07, *P*<0.001) and a significant PrePost × group interaction (*F*(1,28) = 6.40, *P*= 0.017). Paired *t*-tests were subsequently conducted as *post hoc* analyses to investigate the PrePost × group interaction. The control group demonstrated a non-significant increase in structured span score (*P* = 0.115), however, the training group significantly improved in structured span score following training (*P*<0.001). This equated to a mean difference in change score (post–pre) on the structured span between the groups of 0.55 (95% CI 0.11–1.00, *r*= 0.42). Analysis of random trials revealed a significant main effect of PrePost (*F*(1,28) = 13.025, *P*= 0.001) but no other significant main effects or interactions.

Secondary behavioural outcomes were performance on random and structured versions of the spatial span task, and scores on measures of general cognitive function, episodic memory and executive function. Repeated measures ANOVA of spatial span scores, demonstrated no significant main effects of PrePost or group. The main effect of chunking was significant (*F*(1,28) = 24.044, *P*<0.001), with participants performing significantly better on structured compared with random trials. There were no significant interactions between PrePost and group or PrePost × chunking × group, indicating no significant transfer effects of training to spatial span.

**Fig. 2:**
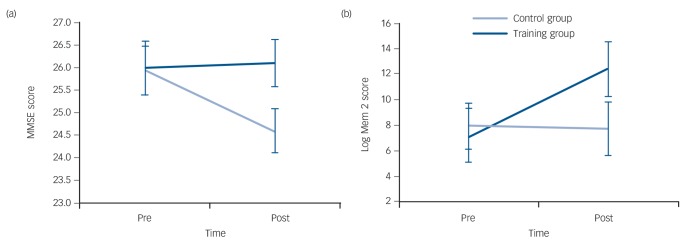
Mean scores pre- and post-interventions on (a) Mini-Mental State Examination (MMSE) and (b) Logical Memory Task 2 (Log Mem 2) tasks. Error bars are standard errors of mean. Time × group interactions are all significant (MMSE *P* = 0.011 and Log Mem 2 *P* = 0.003).

Repeated measures ANOVA examining MMSE score revealed a significant main effect of PrePost (*F*(1,28) = 5.467, *P* = 0.027) and a significant interaction between PrePost and group (*F*(1,28) = 7.383, *P* = 0.011). Results are shown in Table 1 and Fig. 2.

The ADAS-Cog data were not normally distributed, therefore post–pre change in ADAS-Cog scores were calculated for each participant and a Mann–Whitney *U*-test was conducted. This revealed a significant difference between the groups (*U* = 36, *z* = −3.175, *P* = 0.001 (2-tailed)). Related sample Wilcoxon rank tests were therefore performed for each group. The control group demonstrated a non-significant increase in ADAS-Cog score (*z* = −1.412, *P* = 0.158), reflecting a deterioration in cognitive function, while the training group significantly decreased in score (*z* = −2.670, *P* = 0.008), representing an improvement in cognitive function following training.

Repeated measures ANOVA of logical memory score revealed a significant main effect of PrePost (*F*(1,28) = 4.516, *P* = 0.043), and no significant main effect of group. There was a significant interaction between PrePost and group (*F*(1,28) = 10.506, *P* = 0.003), demonstrating a significant training-related improvement in verbal episodic memory function (Fig. 2). The PAL data were not normally distributed. Therefore, post–pre change in PAL scores were calculated and a Mann–Whitney test was conducted, which revealed no significant difference between the groups (*U* = 71.5, *z* = −1.783, *P* = 0.075 (2-tailed)).

Individual repeated measures ANOVAs, with time as the within-participants variable and group as the between-participants variable were conducted on the fluency, grammatical reasoning and odd one out tasks. There were no significant main effects or interactions on any of the executive function tasks. Similarly, Mann–Whitney tests, with post–pre change score as the test variable revealed no significant differences between the groups on the trails A or self ordered search tasks. The trail making test part B data were not analysed due to floor effects, as 18 participants failed to complete the task.

A fixed five-span digit span task was performed in both pre- and post-intervention fMRI sessions. Repeated measures ANOVA demonstrated a significant effect of chunking on performance (*F*(1,28) = 6.871, *P* = 0.014). Both groups correctly recalled more structured than random trials at both pre- (72.1% structured trials correct *v.* 68.6% random trials correct) and post-intervention (76.3% *v.* 71.6%). There were no other significant main effects or interactions.

The fMRI analyses followed an *a priori* region of interest (ROI) approach based on the hypothesis that training-related improvements would be accompanied by plasticity in the functional activity of PFC and parietal cortex areas involved in the task. ROIs were defined as 10 mm spheres around central coordinates in the right dorsolateral prefrontal cortex (DLPFC) (39, 43, 33), left DLPFC (−39, 36, 36), right parietal cortex (46, −40, 42) and left parietal cortex (−37, −45, 37). The parameter estimates produced from each of the factors in the model were summarised across all voxels within each ROI and these values were entered into a repeated measures ANOVA. Within-participant factors were PrePost (pre *v.* post), chunking (structured *v.* random), ROI (DLPFC *v.* parietal cortex) and hemisphere (right *v.* left), with group as the between-participants factor. There were no significant main effects, however, there were significant complex interactions between PrePost × ROI × hemisphere × group (*F*(1,27) = 4.232, *P*= 0.049), PrePost × chunking × ROI × hemisphere (*F*(1,27) = 6.989, *P*= 0.013), PrePost × chunking × hemisphere (*F*(1,27) = 5.422, *P*= 0.028) and a near significant overall interaction between PrePost × group (*F*(1,27) = 3.899, *P*= 0.059). To further determine the basis of these interactions, separate repeated measures ANOVAs were conducted for each trial type. For structured trials there was a significant interaction of PrePost × group (*F*(1,27) = 5.403, *P*= 0.028), a significant PrePost × ROI × hemisphere × group interaction (*F*(1,27) = 5.030, *P* = 0.033), and no other significant main effects or interactions. For random trials there was a significant ROI × hemisphere interaction (*F*(1,27) = 4.562, *P*= 0.042), and no other significant main effects or interactions. To further examine the significant training effects for structured trials further ANOVAs were conducted for each ROI individually.

**Fig. 3:**
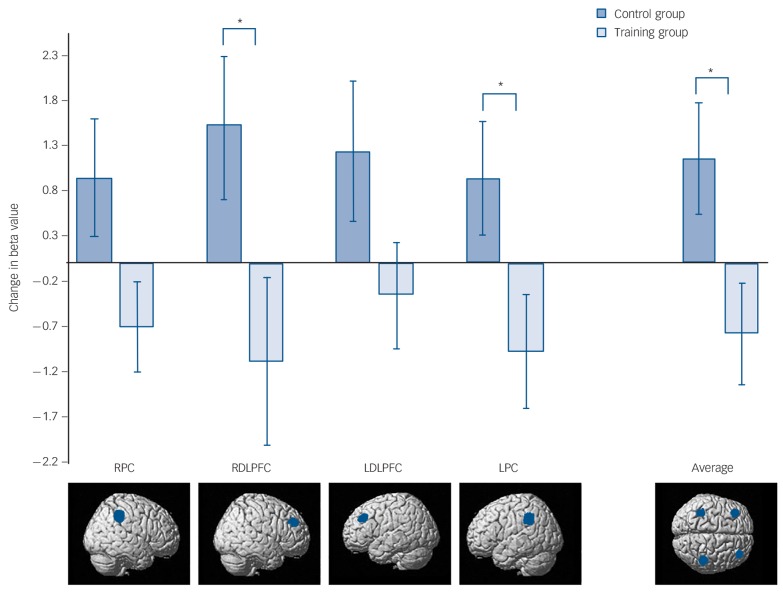
Results of the change in functional magnetic resonance imaging (fMRI) response (calculated as post-beta value – pre-beta value), in each of the four specified regions of interest. Right parietal cortex (RPC, 46, −40, 42), right dorsolateral prefrontal cortex (RDLPFC 39, 43, 33), left dorsolateral prefrontal cortex (LDLPFC −39, 36, 36), left parietal cortex (LPC −37, −45, −37), Average, change in beta values averaged across all four regions of interest. Error bars are standard errors of mean. *group difference significant at *P*<0.05.

In the right DLPFC there was a significant PrePost × group interaction (*F*(1,27) = 4.422, *P* = 0.045), but no main effects of PrePost or group (Fig. 3 and online Fig. DS3(a)). In the left DLPFC there were no significant main effects and no significant effect of training (PrePost × group interaction (*F*(1,27) = 2.735, *P* = 0.110). In the left parietal cortex there was a significant PrePost × group interaction (*F*(1,27) = 4.604, *P* = 0.041) and no significant main effects. In the right parietal cortex the PrePost × group interaction neared significance (*F*(1,27) = 4.072, *P* = 0.054) and there were no significant main effects of PrePost or group (Fig. 3 and online Figs DS4(a) and (b)). These results all demonstrated a similar pattern of training-related reduced activation in all four ROIs as a result of training compared with increased activation in all four regions in the control group. However, the reliability of the effects varied significantly across the frontal and parietal ROIs (Fig. 3).

Whole brain analyses were also conducted to identify any additional voxels or voxel clusters that significantly changed in level of activation, other than the defined ROIs. Examining for training effects (PrePost × group, and PrePost × chunking × group contrasts), revealed no additional significant voxels when corrected for multiple comparisons using family-wise error during either encoding or delay.

## Discussion

### Main findings

This randomised controlled trial demonstrated that 18 sessions of adaptive working memory training in verbal chunking strategies given to patients with Alzheimer's disease, led to significant performance improvements on the trained verbal working memory task, and also to improvements on general cognitive and verbal episodic memory outcomes. Further, the observed cognitive benefits were accompanied by evidence of change in the functional activity of cortical networks that are known to be involved in the task. Although training led to improvements compared with the control group on the MMSE and ADAS-Cog (both clinical measures of general cognitive function), there was no transfer of benefits to non-verbal working memory or episodic memory tasks, or to specific measures of executive function. This apparent discrepancy can be resolved if it is assumed that there was no improvement in intelligence or executive function *per se* as a consequence of training. Instead, training on this digit-based chunking task may have only increased the application of verbal recoding strategies in other tasks. Indeed, generalised benefits were only seen on those tasks that were amenable to the adaptive use of similar chunking strategies. For example, both the story recall in the logical memory task and word recall lists of the ADAS-Cog could potentially be chunked, based on linguistic or verbal-semantic links between test items.

### Interpretation of fMRI results

The consistent pattern of training-related plasticity seen in the brains of participants was of a decrease in functional activity in all ROIs following training. This was in contrast to increased activation in all examined ROIs in control participants between the baseline and follow-up scans. This pattern of results is consistent with a growing literature reporting that cognitive training leads to a decrease in cortical functional activity.^18^ Initially a task may require large attentional and executive resources in order to be successfully performed. This executive resource may be underpinned by a ‘scaffold’ of cortical regions, including the PFC and PPC.^18,19^ As training continues, the requirement for attentional and executive resources diminishes, and therefore activation in this network correspondingly decreases as the ‘scaffolding falls away’.^18,19^ A number of potential neurobiological mechanisms at the synaptic, neuronal and neural network level may be important in such ‘scaffolding’ and efficiency.

Several studies examining the effects of practice have described a decrease in cortical activation within the course of a single session.^18,20^ This suggests that functional redistribution can occur over short timescales of around an hour. However, in keeping with the current study, other studies have also demonstrated similar redistribution dynamics that developed over longer training periods of several weeks.^6^

The initial increase in activation in the PFC–PPC network predicted by the scaffolding/efficiency theory may also explain the results observed in control participants. The active control intervention was a low-level cognitive demand task of only three digits. Therefore, the five-digit span task performed in the scanner would represent a considerable increase in task difficulty from the control intervention. In keeping with this theory, control participants would require increased executive resources to perform the five-digit task, which would be reflected in increased PFC–PPC activity, in contrast to the participants in the training group, who had been adaptively trained. However, while this may explain an increase in activity in the PFC–PPC in untrained participants performing the working memory task, the observation that the activation in control participants increased from baseline to follow-up, rather than remaining at a constant level, needs to be explained. It has been observed that, in line with the efficiency theory, activation in PFC and PPC areas may follow an inverted U-shaped quadratic function, with activity increasing early in training, prior to decreasing.^21^ It is possible that control participants, due to the low-level training they had received, were still at a point near the top of the inverted U-shaped curve, and that adaptive training led to participants in the training group being much further along the curve, so that decreasing activation was observed.^21^ It may also be possible that the increase in activity reflects the improved span performance seen in controls, as they may have been more engaged and trying harder at the task at follow-up compared with their baseline exposure to fMRI.

Critically, however, the current study provides evidence of the potential for functional plasticity following training in an Alzheimer's population. Functional plasticity is increasingly reported in older adults and in mild cognitive impairment;^22^ however, the extent to which training-related plasticity may be possible in Alzheimer's disease remains unclear. These findings provide important and encouraging support for the presence of continued plasticity in the early stages of dementia.

### Strengths and limitations

Limitations of this study include the small number of participants, and it is possible that the study was underpowered to find further areas of significant improvement or plasticity on fMRI. The study, although randomised and well controlled, was not blinded. Therefore, observer bias may be present in the behavioural outcome measures, although attempts were made to avoid this through the use of computerised tasks and validated outcome measures.

This study also demonstrated that computerised working memory training was acceptable to participants with Alzheimer's disease and their carers. Once training had commenced, no participants dropped out of the study, despite the considerable commitment required. Anecdotally, participants enjoyed engaging with the training and control interventions and felt empowered that they were investing in a potentially useful exercise. This reflects the significant public and patient interest in cognitive training, which has become a billion dollar industry; however, there remains a clear need for the efficacy of cognitive training tools to be assessed in well-controlled randomised trials in patients with Alzheimer's disease. The observed benefits of adaptive chunking training in the verbal domain should be broadened in future studies focusing on exploring the utility of this method. For instance, benefits of strategy training could be tracked in the longer term, and similar training in other domains such as object- or spatial-based working memory could be explored. The ability of patients with mild Alzheimer's disease to access and engage with computerised cognitive training tools presented online also needs further investigation, as this approach would enable cognitive training to be made widely available in a cost-effective manner.

Worldwide, in 2013 there were 44.4 million people with Alzheimer's disease, projected to rise to 135.5 million by 2050.^23^ Any tool that could help this very large and highly disabled clinical population would have a profound positive effect on society. We have here described one such potential tool, chunking-based cognitive training, which could be an effective future technique to help maintain cognitive function in early Alzheimer's disease.
